# Cell Behavioral Dynamics as a Cue in Optimizing Culture Stabilization in the Bioprocessing of Pluripotent Stem Cells

**DOI:** 10.3390/bioengineering9110669

**Published:** 2022-11-09

**Authors:** Naruchit Thanuthanakhun, Mee-Hae Kim, Masahiro Kino-oka

**Affiliations:** 1 Department of Biotechnology, Graduate School of Engineering, Osaka University, 2-1 Yamadaoka, Suita 565-0871, Osaka, Japan; 2 Research Base for Cell Manufacturability, Graduate School of Engineering, Osaka University, 2-1 Yamadaoka, Suita 565-0871, Osaka, Japan

**Keywords:** pluripotent stem cells, cell behaviors, culture microenvironments, stem cell bioprocessing, Waddington’s epigenetic landscape

## Abstract

Pluripotent stem cells (PSCs) are important for future regenerative medicine therapies. However, in the production of PSCs and derivatives, the control of culture-induced fluctuations in the outcome of cell quality remains challenging. A detailed mechanistic understanding of how PSC behaviors are altered in response to biomechanical microenvironments within a culture is necessary for rational bioprocessing optimization. In this review, we discuss recent insights into the role of cell behavioral and mechanical homeostasis in modulating the states and functions of PSCs during culture processes. We delineate promising ways to manipulate the culture variability through regulating cell behaviors using currently developed tools. Furthermore, we anticipate their potential implementation for designing a culture strategy based on the concept of Waddington’s epigenetic landscape that may provide a feasible solution for tuning the culture quality and stability in the bioprocessing space.

## 1. Introduction

Pluripotent stem cells (PSCs), which can be isolated from embryos as embryonic stem cells (ESCs) or reprogrammed from somatic cells as induced pluripotent stem cells (iPSCs), are remarkable for their unlimited self-renewal in vitro and ability to differentiate into all cell types of the three embryonic germ layers, including the ectoderm, mesoderm, and endoderm [1]. These cellular capacities make them highly attractive candidates for regenerative medicine. They have been investigated for various clinical complications associated with globally high morbidity rates, including age-related macular degeneration, neurological disorders, and type 1 diabetes [2,3,4]. Despite an extensive exploration of the therapeutic potential of PSCs, there remain challenges toward real-world implementation of these cells in terms of cell bioprocessing and manufacturability.

**Figure 1:**
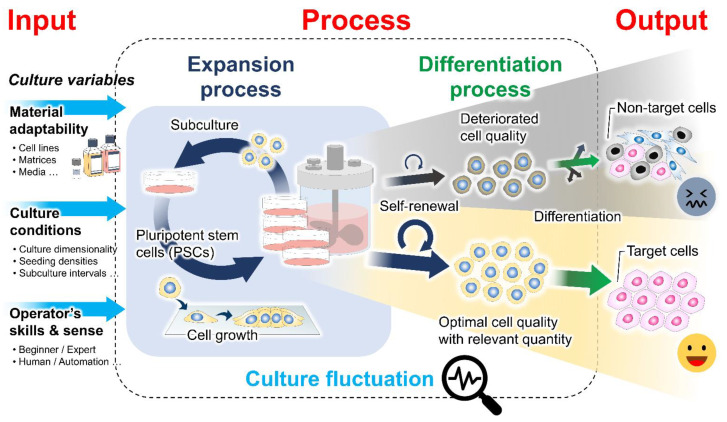
Culture-induced fluctuation in the cell preparation bioprocess. The expansion process contributes to producing an adequate quantity of undifferentiated cells with stable quality for further differentiation and clinical translation. The existence of cell quality variability has been recognized to be influenced by intrinsic cellular properties and extrinsic variables in the cell culture, including raw material adaptability, culture conditions, and the operator’s skills and sense. Differences in the culture inputs and spatiotemporal changes in the cell structural organization during culture determine the biomechanical properties of the cellular microenvironments. The interactions between cells and their microenvironments may result in fluctuations in the phenotypic features of the PSC products.

Cell-based therapies require a high cell density for downstream differentiation and transplantation. Therefore, the development and optimization of robust, scalable, and cost-effective bioprocessing systems are needed to provide an adequate quantity of cells with stable quality [5,6]. Different cell culture systems have emerged for propagating and differentiating PSCs to generate various target cell types. However, the cell variability and suboptimal quality observed within and between batches reflect the existence of fluctuation in the bioprocessing of PSCs (Figure 1) [7,8,9,10]. Typically, the variation in cell quality has been identified to be dependent on the cell origin (origin-induced) and the culture environment (culture-induced) [11]. During in vitro culture adaptation, PSCs accumulate epigenetic changes and potentially undergo phenotypic transition, influencing their clonal self-renewal and lineage differentiation propensity [9,12,13]. It has been shown that variable input factors applied to the culture process, including raw culture materials, culture conditions, and operational parameters, determine the actual microenvironments of the growing cells and how they behave in culture [14]. The dynamic alteration of the cell–microenvironment interactions can profoundly impact many intracellular biological events and cell pluripotency features [10,15,16]. Previously, cellular characteristics and functional variation were investigated among culture platforms. The growth-dependent self-organization within cell colonies under two-dimensional (2D) culture conditions is associated with inter- and intra-colony heterogeneity and spatial differences in the transcription of genes involved in cell cycle regulation, pluripotency, and epithelial function [17,18,19]. The distinct patterns of cell behaviors and growth phase transition during expansion have been further linked with the subsequent preferential differentiation [18,20]. Moreover, in a three-dimensional (3D) aggregate culture system, the growth and survival of PSCs, as well as the cell aggregate structural integrity, are variable, depending on their intercellular interactions and endogenous extracellular matrix (ECM) secretion capability [21,22,23]. After prolonged expansion, the increasing local heterogeneity in ECM accumulation and cytoskeletal alignment, in concert with the uneven distribution of nutrients and essential biochemicals, likely exacerbates growth limitation and spontaneous cell differentiation within 3D cell aggregates [22,24,25]. These prior studies demonstrated that multifactorial variables over courses of culture processes play a role in modulating cell behaviors and regulating the PSC properties and potential. From the perspective of bioprocess engineering, how to efficiently stabilize this fluctuation during PSC culture has become a key challenge in boosting process performance and final cell product quality.

In this review, we aimed to underscore the crucial role of cell behavioral regulation in minimizing the culture-induced fluctuation in PSC bioprocessing. Firstly, we address recent advances in understanding mechanisms by which the cell behavioral dynamics in response to culture microenvironments act to reorganize intracellular mechanics and orchestrate the stem cell states and potential. Next, we delineate how to regulate the cell behaviors using developed culture tools and culture microenvironments to modulate the cell mechanical homeostasis and pluripotency functions during culture. Finally, we discuss principal considerations in culture strategy design for tightly controlling the PSC expansion and derivation processes based on the conceptual Waddington’s epigenetic landscape.

## 2. Mechanistic Roles of Cell Behavioral Dynamics in Modulating Cell Fate Decision

**Figure 2:**
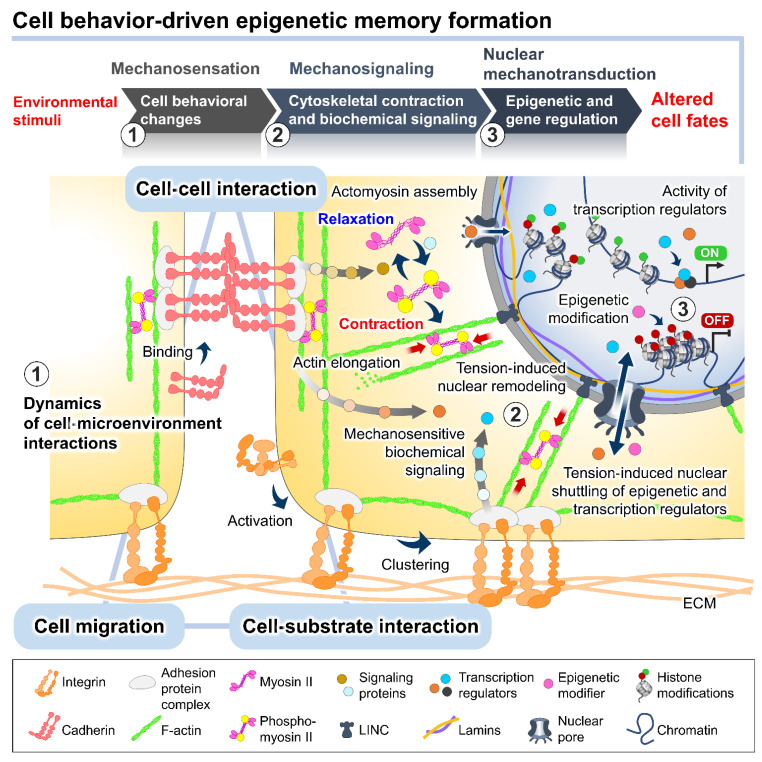
The plausible interplay between cell behavioral dynamics and intracellular mechanical regulation in cell fate modulation. In the culture microenvironments, cells sense and respond to extrinsic mechanical stimuli from the culture substrate, surrounding cells, and fluid dynamics exerted on cellular membranes and different biomechanosensors, such as integrins and E-cadherins. The alteration of the integrin-mediated cell–substrate and E-cadherin-mediated cell–cell adhesion behaviors triggered by the extrinsic forces can stimulate a wide range of intracellular biochemical signaling cascades and induce intrinsic force generation by regulating the actomyosin cytoskeletal contractility. The distribution of the intrinsic cytoskeletal tension mediates the nuclear structural remodeling and cytoplasmic-nuclear shuttling of several mechanosensitive transcription regulators, influencing the intranuclear events, such as epigenetic modifications and gene transcriptional activity, and consequently altering cell fates and functions.

Recent evidence has indicated that complex reciprocity between cells and culture microenvironments produces dynamic changes in cell behaviors and intracellular mechanics, contributing to the spatiotemporal variations in cell state and functional regulation within culture [26,27,28,29]. In addition to biochemical cues, cultured cells continually perceive extrinsic stimuli through direct interactions with their surroundings and relay them intracellularly to regulate the multitudes of molecular pathways and gene transcriptional networks. This process is called mechanotransduction (Figure 2) [28,30]. At the cell surface, the anchorage of cells to the local ECM components and the neighboring cells, mediated by specialized adhesion molecules, such as integrin and cadherin, conjointly exerts a mechanosensation role and coordinates cell migration [31,32,33]. The strength of the cell adhesion interactions depends on the magnitude of mechanical constraints sensed by the cells, eliciting a graded mechanosignaling output from the adhesion sites [34,35].

The binding of cell adhesion molecules promotes the recruitment of multiple structural and signaling proteins at the cytoplasmic domains. The association of adhesion protein complexes primarily facilitates a direct force transmission from adhesion contacts towards internal cell compartments and nuclei via physical cytoskeletal connections [36,37]. Moreover, the exerted forces on cell adhesion can be converted into biochemical signals by altering the compositions and activities of regulatory proteins at the adhesion complexes, which generates a sequence of signaling pathways and changes in the cytoskeletal alignment and tensional dynamics [38,39]. The adhesion-mediated activation of Rho A, a member of the Rho GTPase family, and ROCK signaling promotes the phosphorylation of motor protein myosin II and inhibits the activity of myosin II phosphatase [40,41]. The kinetics of the assembly between the phosphorylated myosin II and filamentous actin termed actomyosin complex produces alternating cycles of actin cyto-skeletal contraction and relaxation [42]. Active Rho protein at cell–cell adherens junctions produces signals through its effectors to establish apical actomyosin networks [17,38]. Whereas the localization of p120-catenin elicits the recruitment and activation of other Rho GTPases, Rac and Cdc42, inducing actin polymerization and suppressing the Rho/ROCK-dependent actomyosin contractility [43]. The antagonism of the Rho GTPase members in controlling contraction or elongation of actin bundles serves as a molecular switch to manipulate the balance between cell adhesive and migratory behaviors and direct the intra- and intercellular tensional homeostasis.

The actomyosin-induced contraction propagates mechanical cues to the nucleus, which are directly transmitted to the chromatin domains via a linker of nucleoskeleton and cytoskeleton (LINC) complexes and further modulates the cytoplasmic-to-nuclear translocation of epigenetic modifiers and transcription regulators [27,44,45]. The direct tethering of actomyosin to the nucleoskeleton and associated chromatin influences the dynamics of nuclear deformation and nuclear lamina–genome interactions based on increased or decreased actomyosin activities [46]. Studies demonstrated that actomyosin-induced nuclear flattening and chromatin condensation in the mechanical stress-applied cells involve the alteration of histone methylation and the transcriptional upregulation of mechanoresponsive and quiescent genes [27,47,48]. 

Moreover, changes in actin polymerization and contractility were implicated in the spatial redistribution of histone deacetylases, leading to global changes in histone acetylation and the switching of gene-expression patterns in cells under different geometric constraints [49]. The cellular tension has also been shown to regulate the nuclear shuttling of mechanosensitive transcription regulators, such as YAP/TAZ and β-catenin, thus controlling the transcriptional activity of their target genes [50]. Current evidence elucidates that the YAP/TAZ nuclear translocation and epigenetic modifications might serve as possible pathways through which the culture-induced cell mechanical tension could affect the self-renewal and fate potential of PSCs [20,51,52,53]. Mechanistically, the nuclear YAP can bind to and stimulate the transcription of diverse pluripotency-associated genes responsible for maintaining pluripotent states and cell proliferation [54,55]. Strikingly, YAP-occupied genes were found to overlap highly with the binding targets of the core pluripotent transcription factors OCT3/4, NANOG, and SOX2, highlighting their collaborative role in tuning the PSC properties [54]. In addition, the nuclear availability of YAP was found to be associated with histone H3 modifications and the formation of site-specific transcriptional enhancers, which dictate the lineage differentiation of PSCs [56]. Furthermore, a transition between the two different states of pluripotency in vitro, commonly referred to naïve and primed states that reflect different stages of embryonic development in vivo, can be influenced by the nuclear-cytoplasmic shuttling of β-catenin protein [57,58]. The retention of cytoplasmic β-catenin promotes the self-renewal of ESCs in a primed pluripotent state through interaction with cytoplasmic TAZ [57]. The activation of β-catenin maintains ESCs in the naïve pluripotent state, while its inhibition induces a naïve-to-primed conversion [58]. The complex networks of transcription factors and epigenetic regulators play a central role in controlling cellular pluripotency. These studies manifest a range of pivotal intracellular elements that ultimately mediate the intranuclear modulation of pluripotency-associated gene regulation. Multistep mechanotransduction processes from the cell membrane to intranuclear architectures elucidate how cell behavioral mechanics could regulate gene transcription and cell phenotypic characteristics. 

**Figure 3:**
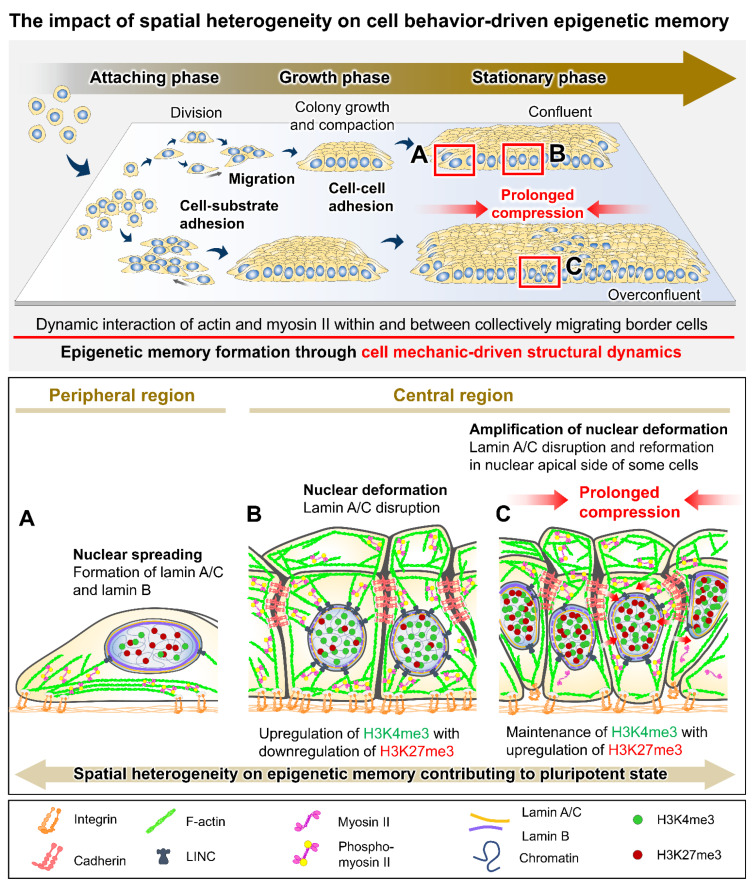
The impact of spatial heterogeneity on cell behavior-driven epigenetic memory. Position- and growth-dependent changes in cell behavioral characteristics during colony growth and compaction in 2D monolayer culture (**A**–**C**) [17,18,24,68]. The differences in cell behavioral mechanics between the cells in different regions and different compressional constraints could distinctly induce the reorganization of the actomyosin cytoskeleton and nuclear laminas, leading to differences in nuclear structural deformation and epigenetic modifications [20,24]. The local heterogeneity within growing cell colonies ultimately contributes to the spatial transcriptional regulation of pluripotency-associated genes and possibly triggers deviation from the pluripotent state [17,19,67,68].

Perturbations in the cell adhesion balances and tensional homeostasis of the PSCs by either intrinsic or extrinsic factors could modulate the molecular circuitry of pluripotency and lineage-specifying signals, influencing stem cell features, such as pluripotent state transition, lineage differentiation preference, and spontaneous differentiation [26,27,59,60]. In addition, changes in cellular mechanics, intracellular circuits, and epigenetic information might be sustained over time and stored as memory, which could affect cell behavioral adaptability and fate decision during prolonged culture or after the cells move to a new environment [9,20]. Prior research demonstrated that functional inhibition of integrin α6 could cause a notable reduction in the core pluripotent gene expression while blocking integrin β1 results in the priming of ESCs for a mesodermal fate [61,62]. The dysregulation of cell–substrate and cell–cell adhesion in PSC colonies has induced abnormality in actomyosin formation and contraction by inducing the Rho/ROCK signaling cascades [63,64]. The impaired actomyosin contractility decreases cell colony integrity and YAP activity, causing defects in pluripotency maintenance and cell survival [15,40,41,52]. During colony formation and compaction in a 2D culture of PSCs (Figure 3), cell adhesion and cytoskeletal reorganization occur in response to spatiotemporal changes in cellular conformation and cell–microenvironment interactions. These changes correlate with the dynamic modifications of global histone methylation H3K4me3 and H3K27me3, which represent key active and repressive marks acting in concert to regulate the epigenetic states of pluripotent and developmental genes [20,65]. Within cell colonies, cell positions at the peripheral and central regions show differences in kinematics and mechanical dynamics, including the apical actomyosin reorganization and nuclear structural deformation, consequently inducing distinct spatial transcription and protein expression of cell adhesion and pluripotency-associated markers [17,18,66]. In prolonged culture beyond confluence, iPSCs exhibit integrin downregulation, apical actin disorganization, and an increase in global H3K27me3 levels; moreover, some cells within densely packed colonies show the reformation and alignment of nuclear lamin A/C, contributing to changes in nuclear stiffness [20,67]. Nuclear lamina remodeling in association with the anomalous cell migration and imbalance between cell–cell and cell–substrate interactions in PSC colonies might trigger a switch from the undifferentiated to a deviated state [68]. Moreover, a distinct mechanical tension at the outer rim of the cell colony was shown to direct fate decision-making events upon differentiation [69,70]. For example, during the early mesendoderm induction in ESC culture, the physical connection between E-cadherin, β-catenin, and the actin cytoskeleton is responsible for mechanosensation and Wnt signaling restriction to the colony boundary, particularly positioning mesendoderm formation [71].

**Figure 4:**
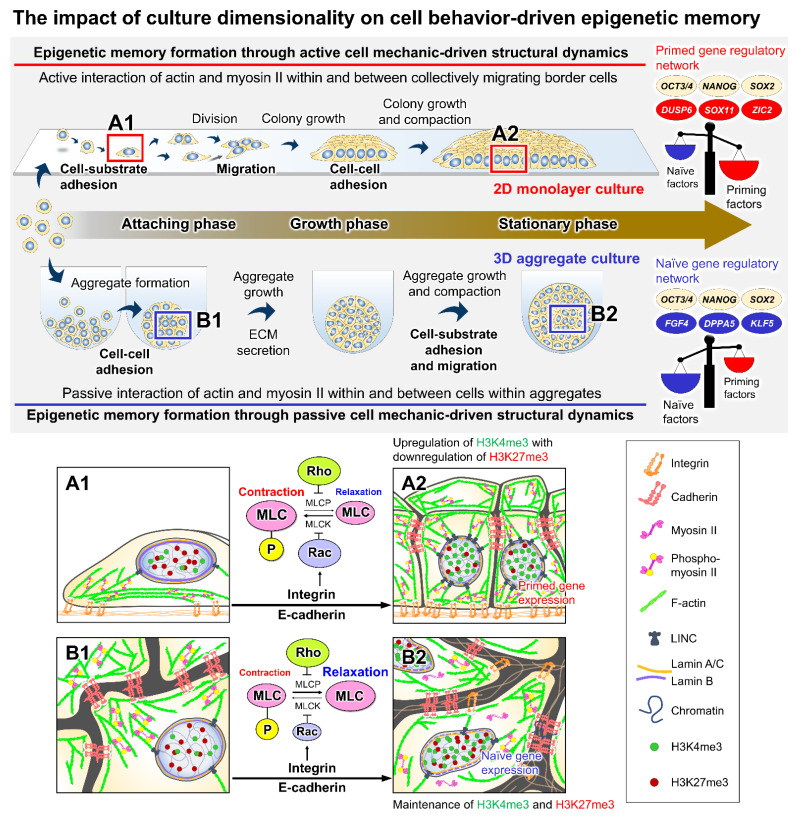
The impact of culture dimensionality on cell behavior-driven epigenetic memory. Cells adapt to different 2D and 3D culture conditions by modulating cell adhesion interactions and the balance of Rac/Rho GTPase antagonism [21,24,73]. In contrast to cells in 2D monolayers (**A1**,**A2**), cells in 3D aggregates interact with the surroundings in all dimensions and modify the actomyosin cytoskeletal contractility by altering myosin phosphorylation activity (**B1**,**B2**). The culture dimensionality and changes in force distribution within 3D structure potentially influence the maintenance of epigenetic memory and the distinctive transcriptional expression of naïve pluripotency-associated genes, consequently attuning the cellular pluripotency [24,74,78].

Compared with the cells in 2D culture, cells in 3D aggregate culture embrace a com-plex regulation of cell–cell and cell–ECM adhesion along the three dimensions of interactions and stimulate their endogenous ECM secretion (Figure 4) [21,72]. Emergent self-organization in 3D aggregate structure causes the radial organization of the ECM and actomyosin bundle alignment, which have been suggested to incorporate outside–inside force transduction throughout the spheroid structure [24,66,73]. iPSC aggregates displayed strikingly different time-dependent cell adhesion protein expression profiles and moderation of myosin phosphorylation compared with the cells in 2D culture, indicating the relaxation of cytoskeletal tension in a 3D culture [24]. During growth and expansion, the cells in 3D aggregates remarkably kept both H3K4me3 and H3K27me3 at high levels and maintained the expression of core pluripotency-associated genes. Interestingly, in response to the 3D environment, the cells stimulate the transcriptional upregulation of naïve pluripotency-associated genes and the downregulation of primed genes, which might be interrelated with their distinct epigenetic memory and the modulation of mechanosensitive YAP signaling pathways [24,74,75]. Furthermore, several studies demonstrate that the directed differentiation of PSCs within the 3D aggregate culture platform could enhance the differentiation and maturation efficiency of various target cell types, such as beating cardiomyocytes and insulin-producing pancreatic cells [76,77]. These studies show that mechanical asymmetry arising from growth-dependent structural reorganization and culture dimensionality locally regulates cell behaviors and intracellular tensional forces along the culture duration, leading to a spectrum of cellular responses ranging from immediate changes in cytoskeletal contractility to long-lasting epigenetic and transcriptional gene modulation [17,18,24]. The effects of cell behavioral alteration induced by several other culture variables need further clarification. How to control the homeostasis of cell behavioral and mechanical regulation among cell populations should be considered when developing and optimizing the culture processes of PSCs.

## 3. Emerging Methods for Enhancing PSC Expansion through the Regulation of Cell Behaviors

Spatiotemporal differences in cellular microenvironments and structural self-organization along the culture of PSCs potentially contribute to cell-to-cell phenotypic variability [14,16]. Fine-tuning three key components of cell mechanical transducers at the cell-microenvironment interface (cell–substrate interaction, cell–cell interaction, and cell migration) by applying alternative culture substrates and biochemical molecules has been recently introduced by several groups (Table 1) [35,67,79,80,81]. The interaction of PSCs with their surrounding ECM plays a role in coordinating the balances of force generation at the cell-ECM contacts and the overall strength of the intracellular and intercellular contraction [27]. Recent advancements in the field of biomaterials provide a wide range of surface coating agents, including recombinant ECM proteins and synthetic biomimetic matrices [35,82]. Differences in the biochemical composition, molecular structure, and mechanical characteristics of culture matrices strongly influence cell behaviors and pluripotent capacity [53]. The distinct structural isoforms of the ECM adhesion molecules, such as laminin-511, -521, -332, -211, and -111, have been shown to affect the efficiency of proliferation and differentiation of PSCs [53,83,84]. Previous research demonstrated that E8 fragments of laminin-511 and -332, which are the minimal forms retaining the integrin-binding specificity, successfully maintained iPSCs in an undifferentiated state with a normal karyotype and pluripotency for more than 30 passages [83]. However, differences in the binding affinity to E8 fragments of laminin-511, -332, and -211 determine the degree of cell colony compaction and actomyosin contractility, consequently switching the differentiation propensity of cultured iPSCs towards distinct ocular lineages involving Wnt and YAP signaling modulation [53]. 

The availability of synthetic polymer- and peptide-based matrices with tunable mechanical properties allows users to regulate an optimal strength of cell–substrate adhesion in a target cell-specific manner, thereby promoting an efficient generation of desired cells [35,79,85,86,87,88]. Stiffness represents a key mechanical property of coating material, critically dictating the subcellular allocation and activity of the integrin-mediated adhesion molecules, and further modifying the cell interactions with neighboring cells [89]. Cultivating ESCs on tunable decellularized fibroblast-derived matrices indicated that the extent of substrate stiffness modulates their cell–substrate adhesive potential and cell motility, mediating either induction or inhibition of the epithelial to mesenchymal transition program and controlling the activity of pluripotent gene expression [28]. The ranges of optimal stiffness should be considered when developing culture matrices to facilitate pluripotency maintenance and long-term cell expansion [90]. Recent research on synthetic hydrogel systems has introduced a concept of cell behavioral control through the in situ modifying of structural and adhesive microenvironments [91]. Combined hydrogel matrices have been optimized to switch between pluripotency-permissive and differentiation-permissive states via ionic de-cross-linking [91]. Interestingly, controlling the timing of matrix switching can regulate the ESCs to differentiate into ectoderm or mesendoderm lineages [91]. These culture matrices have been used successfully to generate an integrated platform for growing undifferentiated ESCs and subsequently differentiating them into terminally specialized cells [91]. Additionally, hydrogel-based matrices have been applied to fabricate labile substrates with patterned islands, which restrict the cell–substrate adhesion to designated areas and induce the self-assembly cell aggregation for producing size- and shape-controlled 3D cell aggregates [78]. Regulating the aggregation kinetics by adjusting the labile substrate ligand density allows for the controlling of the porous structure of cell aggregates and indirectly determining stem cell fate [78].

The exogenous regulation of integrin- or E-cadherin-mediated adhesion can attune the properties and functions of cultured PSCs [27,31,92]. Applying a uniform mode of integrin- or E-cadherin-based adhesion regulation attenuates the spatial cell heterogeneity in culture [31]. A non-colony culture system based on recombinant E-cadherin-immobilized surfaces has been proposed to grow undifferentiated PSCs in a more homogeneous microenvironments with moderation of cell–cell contacts [93]. The cultures of ESCs and iPSCs on an E-cadherin-coated substrate, which retain their E-cadherin-based interaction, have been found to increase cell proliferation and maintain cell viability and pluripotency during subculture [93,94]. Furthermore, to modulate the cell–cell interaction, the E-cadherin function-blocking agent botulinum hemagglutinin (HA) has been used as a culture tool to selectively remove cells that deviate from an undifferentiated state during the expansion of iPSCs [67]. Due to the weakened E-cadherin-mediated cell–cell adhesion in deviated cells, HA-induced E-cadherin disruption causes the detachment of deviated cells from cell colonies; however, the undifferentiated cells can restore the E-cadherin-mediated cell–cell interaction and retain their pluripotency following HA removal [67]. Moreover, routine HA treatment in serial passages has been shown to facilitate the long-term maintenance of the iPSC population in an undifferentiated state [81]. It has been suggested that the temporal relaxation of cell–cell junctions by HA can stimulate cell migratory behaviors and cytoskeletal rearrangement, resulting in a relatively uniform dispersion of cells in colonies [81]. In addition, the HA-mediated temporal cell–cell adhesion disruption has been adopted to establish an in situ cell aggregate break-up method for high-density suspension expansion [95]. In cell aggregate growth in conventional culture, large-size aggregates enhance collagen type I accumulation on the aggregate periphery, restricting the homogeneous microenvironments and consequently resulting in undesirable cell proliferation and cell necrosis within the aggregate. The HA-mediated dissociation of cell–cell adhesion facilitates the break-up of aggregates into small sizes, allowing a significant increase in the expansion fold of cells with no adverse effect on maintaining pluripotency [95]. These studies represent current progress in tailoring cell behaviors in PSC cultures. The use of emerging culture strategies that integrate the precise control of culture microenvironments and cell behavioral dynamics may ultimately contribute to regulating the maintenance of undifferentiated state and pluripotent ability of cultured PSCs along the expansion process.

**Table 1:** Developed culture tools for PSC expansion and downstream differentiation.

Classification	Tools	References
Laminin-based culture substrates	▪ Full-length laminin-511, -521, -332, -211, and -111	[53,83,84]
	▪ E8 fragments of laminin-511, -332, and -211	[53,83]
Synthetic polymer- and peptide-based culture substrates	▪ PMVE-alt-MA, PMEDSAH, and PVB *	[85,86,87]
	▪ Hydrogels	[35,79,90,91]
	▪ Chitosan-alginate polymers	[75]
	▪ Synthemax™	[88]
E-cadherin-based culture substrates	▪ E-cadherin-Fc chimera	[93,94]
E-cadherin function-blocking agents	▪ Botulinum hemagglutinin	[67,81,95]

* PMVE-alt-MA: Poly(methyl vinyl ether-alt-maleic anhydride); PMEDSAH: Poly [2-(methacryloyloxy)ethyl dimethyl-(3-sulfopropyl)ammonium hydroxide]; PVB: Poly(vinyl butyral).

**Figure 5:**
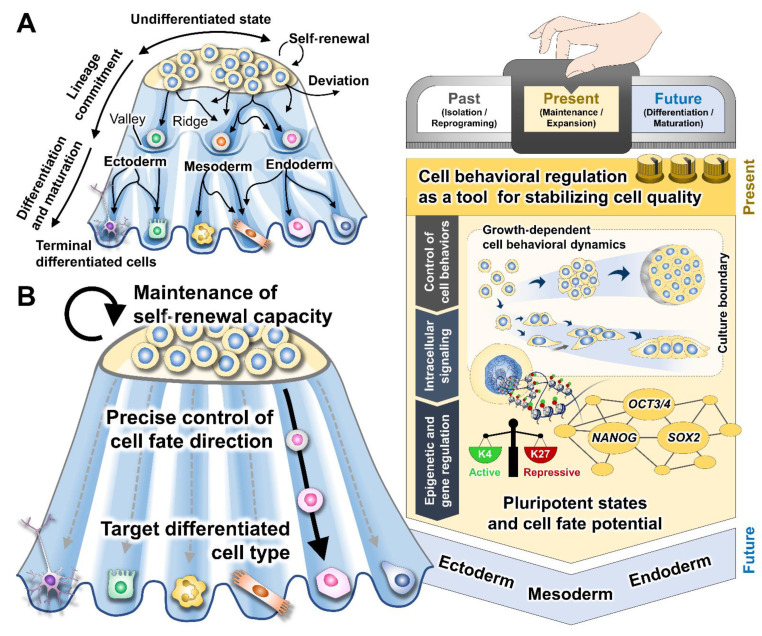
Prospects in designing culture strategy for controlling cell quality based on Waddington’s epigenetic landscape concept. (**A**) Considering Waddington’s landscape, the progression of the PSC fate decision has been depicted intuitively as a cell resting and traveling on a compound metaphor. Valleys on the landscape where the cells are retained imply the states where the cellular potency is maintained. Upon receiving the extrinsic stimuli, cells are induced to move downhill, pass through a cascade of branching ridges and valleys, and finally reach their destinations as specialized cells. The topography of the ridges and valleys, which modulates path choices and fate decisions, is conceptually fine-tuned by a complex interplay between the extracellular stimuli and the intracellular gene regulatory networks. (**B**) Regarding PSC bioprocess engineering, the robust and precise regulation of the undifferentiated state maintenance and cell fate specification is a prerequisite to ensuring the resultant quality of intermediate and final cell products. The current understanding of the impact of cell–culture microenvironment interactions on the modulation of PSC states and fate potential indicates a significance of cell nurturing strategy design. The balance between cell behaviors and mechanics has an important role in orchestrating the intracellular signaling and the epigenetic and transcriptional regulation of pluripotency- and development-associated genes, which mechanistically influence the states and functions of the cultured PSCs. The rational implementation of culture environments and cell behavioral regulation tools may help instruct such intracellular regulatory processes and minimize the occurrence of undesired cells and cell-to-cell variability, eventually facilitating an effective production of PSCs and their derived cells.

By considering the cellular plasticity and regulatory complexity in the stem cell development as visualized by Waddington’s epigenetic landscape (Figure 5), the control of cell behaviors and cell mechanics might be further implemented to direct a cellular transition from an undifferentiated state to a desired cell fate [96,97,98,99,100]. From the top of the rearranged epigenetic landscape, the determination of a specific path conceptually manifests the regulation of cell fate specification and differentiation direction towards a target cell type. It has been denoted that the morphogenetic and molecular status of the initial undifferentiated cells crucially determines the fate decision [98,100,101]. During early differentiation induction, the well-organized transformation of the cell behavioral and mechanical phenotypes is coupled with the responsiveness of cells to biochemical differentiation stimuli in reinforcing the pluripotency exit and fate commitment [102,103]. Conversely, the delay or dysregulation in cell behavioral modulation can result in differentiation resistance and non-target cell generation [39,104]. Manipulation of cell–cell interaction and cellular mechanics has been shown to improve the mechanosensation of cells to the differentiation signals and prime the transcriptional initiation of lineage-specific genes, promoting cell differentiation efficiency [31,71,101]. Altogether, culture approaches that allow optimal interactions between cells and microenvironments might serve as the foundation for developing robust cell culture platforms that can enhance and stabilize the preparation of PSCs and target specialized cells for downstream applications.

## 4. Concluding Remarks and Future Perspectives

Crosstalk between cell behavioral dynamics and intracellular mechanical regulation has been elucidated to incorporate extrinsic cues exerted on the cell–microenvironment interfaces to direct short-term and long-term stem cell phenotypic and functional alterations during culture [39,40,64]. Increasing considerations on cell culture design principles drawn from the association between cell–microenvironment interactions and the stem cell developmental mechanisms have now set the stage for improving the bioprocess preparation of PSCs and derivatives.

To control the PSC quality during culture process, it is critical for bioengineers to comprehend the properties of the cells they are using and to construct optimal in vitro culture environments. The boundary of cell expansion conditions must be determined specifically for each culture system that involves different input variables, such as different culture media and culture platforms, and distinctly influences the patterns of cell behavioral dynamics and growth phase transition [14]. More importantly, throughout the culture period, cellular environmental factors should be quantitatively examined and regulated with standard criteria to prevent a detrimental impact of mechanical and biochemical perturbations on the cells. To evaluate the status of cultured cells, in addition to the routine offline analysis including the examination of positive and negative pluripotency biomarkers, effective in-process monitoring and culture control methodologies are required for tracking the cell behavioral kinetics and minimizing an aberrant alteration of cell behaviors as well as an undesirable emergence of suboptimal cell populations [105,106]. In the translation from small-scale settings to large-scale cell production, a meticulous validation of a redesigned and scale-up process is essential to verify its performance and the stability of cell products. Within recent decades, a variety of culture platforms, such as a multi-layered cell factory, microcarrier-based bioreactors, and cell aggregate bioreactors, have been proposed for large-scale use. Compared with conventional culture, the effect of geometry, substrate, and mechanical dynamics within those culture platforms on the cell behavioral regulation and pluripotency preservation must be thoroughly elucidated.

In addition to bioprocessing considerations, the further exploration of the biological nature of the cultured PSCs, particularly their in vitro-state plasticity and developmental mechanisms, is still needed to bridge remaining knowledge gaps and aid the culture development and optimization. Recent advancements in single-cell multi-omics profiling technologies might help dissect the transcriptomic, epigenetic, and phenotypic heterogeneity of bulk cell populations and promise to deepen the understanding of the intracellular regulatory network associated with the modulation of the PSC state at a single-cell level [107,108]. Along with cell expansion and differentiation, the growing complexity and spatiotemporal dynamics of cell structure regarding the culture dimensionality and stem cell developmental stages are considered to hinder the in-depth investigation of cell behavioral and mechanical kinetics. As such, more integrative and time-resolved analysis tools are necessary. Comprehensive mechanistic insights into the molecular basis of cellular interactions with specific components of the culture microenvironments would open an avenue for identifying the design space in the culture processes of PSCs and their derivatives. The continuous development and improvement of bioprocess systems by harnessing the combined power of biology and engineering may help diminish the culture quality stochasticity and reinforce a shift of PSC technologies from the realm of bench research to the domain of cell manufacturing and clinical uses. This will allow the full exploitation of the therapeutic potential of PSCs.

## Data Availability

Not applicable.
